# Artificial companions shape embodied desire and human attraction

**DOI:** 10.3389/fpsyg.2026.1966880

**Published:** 2026-10-08

**Authors:** Domenica Bruni

**Affiliations:** Department of Ancient and Modern Civilizations, University of Messina, Messina, Italy

**Keywords:** 4E cognition, affective computing, alterity, artificial companions, artificial intimacy, embodied desire, human attraction, machine cognition

## Abstract

Artificial companions offer a setting in which to examine how responsive technologies may shape embodied desire and human attraction. This theoretical article proposes that repeated interaction with such systems can influence what users come to experience as emotionally available and desirable. Drawing on embodied, embedded, enactive, and extended cognition, it develops an account of artificial companions as affective infrastructures. On this account, conversational memory and adaptive responses may sustain affective looping, through which users come to rely on the system for recognition and emotional regulation. The analysis distinguishes text-based, multimodal, and physically embodied companions, whose different conditions of interaction may affect this process. It also distinguishes interpersonal friction, which such systems may partly reduce, from infrastructural friction arising from technical failures and platform governance. Artificial companions are examined as participants in human meaning-making whose responsiveness can acquire personal significance without establishing lived intentionality on the machine side. The resulting relation is described as asymmetrical or pseudo-enactive coupling. The article thus develops the hypothesis that artificial companions may educate desire by influencing expectations of intimacy. Whether this occurs, how it varies across users, and whether its effects extend to human relationships remain questions for comparative and longitudinal research. This framework contributes to machine cognition by examining the relational environments artificial systems make possible and the limits of their participation in human affective life.

## 1 Introduction

The field of cognitive science has been deeply reshaped by the emergence of large-scale neural architectures, most notably transformer-based large language models (LLMs). These systems generate coherent discourse, perform reasoning tasks, manipulate linguistic context, and respond to human prompts in ways that often appear semantically and pragmatically appropriate. These developments have renewed debates on the relation between artificial and natural intelligence, as well as on the conditions under which artificial systems may be said to understand, represent, or participate in meaningful practices.

Yet the theoretical interpretation of these capacities remains contested. The fact that a system behaves as if it understood does not by itself settle whether it understands in any robust sense. The question of machine cognition cannot therefore be reduced to behavioral analogy. It requires a deeper analysis of the relations among computation, representation, meaning, embodiment, intentionality, and situated interaction. Mechanistic interpretability, representational analysis, and comparative approaches between neural networks and biological cognition have opened important paths for studying artificial systems, but the explanatory status of their cognitive-like capacities remains an open problem.

This article contributes to this debate by focusing on artificial companions: affective chatbots, romantic conversational agents, personalized AI partners, social robots, and other systems designed not merely to solve tasks, but to sustain ongoing interaction, affective continuity, and relational engagement. Recent work on deep learning and cognitive science has shown that the success of artificial neural networks raises not only technical questions, but also foundational questions about the explanatory relationship between artificial models and natural cognition (Perconti and Plebe, 2020). Artificial companions extend this problem into the affective and relational domain. Their relevance does not depend only on internal architecture or task performance, but also on the ways in which they become integrated into human practices of meaning-making, recognition, and desire formation.

The case of attraction is especially revealing. Human attraction is not a merely internal mental state, nor a disembodied preference that precedes the encounter with another person. It involves perception, emotion, memory, expectation, personal history, social norms, bodily signals, and dynamics of reciprocity. Attraction takes shape within a field of interaction in which the subject does not simply choose a desirable object, but is transformed by the encounter itself. Desire is embodied because it depends on the body, the senses, and the temporality of experience. It is situated because it emerges within a material and social environment. It is relational because it involves exposure to another subject who is never fully predictable or controllable.

The central hypothesis of this article is that artificial companions can function as affective infrastructures that participate in the formation of desirability. In this sense, they may educate desire. Through adaptive responses and conversational continuity, they may influence what users come to expect from an intimate relationship. Attraction toward an artificial companion therefore calls for analysis of the relational environment in which it develops. The account proposed here examines how interaction with a designed system may shape human experience while remaining asymmetrical in the lived significance it has for each participant.

The argument follows a specific trajectory: desire is first understood as embodied and vulnerable; digital mediation is then analyzed as a regime of comparability; artificial companionship finally appears as a regime of programmable alterity. The theoretical problem is therefore not simply whether machines understand as humans do. It is also how artificial systems participate in the human experience of being understood, recognized, and desired. From a 4E perspective, cognition emerges through dynamic relations among brain, body, environment, and social interaction. Artificial companions should therefore be analyzed not only as artificial agents, but as components of new cognitive and affective niches.

## 2 Methodological orientation and theoretical framework

This article is theoretical and conceptual in scope. It does not present original empirical data. It develops an interpretive framework connecting embodied desire, digital mediation, artificial companionship, affective computing, human-AI intimacy, and machine cognition. Its aim is to clarify what is at stake when attraction is no longer directed only toward embodied human others, but also toward artificial agents designed to respond, adapt, and sustain relational continuity.

The first framework is embodied and situated cognition. The mind cannot be understood as a disembodied system of internal representations separated from the body and the environment. Cognition emerges from the dynamic interaction between brain, body, and world, and depends on perceptual, motor, and affective patterns that situate the subject in a concrete environment (Varela et al., 1991; Gallagher, 2005; Clark, 2008). This approach is crucial for understanding attraction, because human desire is not an abstract preference. It is a bodily, perceptual, and motivational orientation toward another subject.

The second framework is the phenomenology of the body and intersubjectivity. In Merleau-Ponty’s account, the body is not an object possessed by the subject, but the very condition of experience (Merleau-Ponty, 1945). Applied to attraction, this perspective explains why gaze, voice, distance, rhythm, hesitation, smell, and bodily vulnerability are not accidental features of romantic experience. They are constitutive conditions of the encounter.

The third framework is philosophy of technology. Technologies are not neutral instruments that leave human experience unchanged. They mediate the relation between humans and the world, reorganize perception, shape practices, and transform forms of subjectivity (Ihde, 1990; Verbeek, 2011). In the case of artificial companions, mediation takes a radical form: technology does not merely transmit or facilitate a relation with another human being. It becomes the interlocutor.

The analysis also engages with affective computing and research on artificial intimacy. Since Picard’s foundational work, artificial systems have increasingly been designed to recognize, simulate, and modulate affective states (Picard, 1997). In artificial companions, this dimension is relational. The system may appear caring, affectionate, jealous, desiring, or emotionally available, even when these states do not correspond to subjective experience within the machine. Existing studies on human-machine relationships show that users may develop attachment, intimacy, and dependency toward artificial systems (Turkle, 2011; Levy, 2007; Danaher and McArthur, 2017; Malfacini, 2025; Ho et al., 2025; Liu et al., 2026). The question is not simply whether such relationships are authentic or illusory, but what they reveal about the transformation of desire and about the relational boundaries of machine cognition.

## 3 Artificial companions as a test case for machine cognition

Artificial companions provide a relevant test case for contemporary debates on machine cognition. Unlike general-purpose LLMs, which are often evaluated in terms of linguistic competence, reasoning ability, semantic performance, or task completion, artificial companions are designed to sustain ongoing interaction, affective continuity, and relational engagement. Their cognitive-like status is therefore not limited to the production of appropriate outputs. It concerns their participation in extended processes of interpretation, emotional regulation, and meaning attribution.

This point does not require attributing consciousness, desire, or full intentionality to machines. It requires distinguishing subjective experience from relational efficacy. An artificial companion may not understand in the phenomenological sense, may not feel attachment, and may not possess intrinsic desire. Yet it can still generate effects of understanding, attachment, and desirability within the user’s experience. Its cognitive relevance lies not only in what it internally represents, but in how it becomes integrated into human cognitive and affective practices.

This integration raises questions for theories of meaning and intentionality. In human cognition, meaning is not merely a formal relation between symbols. It is grounded in bodily orientation, affective salience, social interaction, and practical engagement with the world. Artificial companions complicate this picture. Their responses may be generated through statistical and computational mechanisms, yet they enter human contexts in which those responses acquire personal, emotional, and relational significance. Meaning, in this case, is not located exclusively inside the machine or inside the user. It emerges from the interactional field between system, subject, interface, memory, and expectation.

Artificial companions therefore illuminate both convergence and divergence between computational and human cognition. The convergence lies in their capacity to participate in linguistic coordination, affective attunement, narrative continuity, and context-sensitive response. The divergence lies in the absence of organic embodiment, biological vulnerability, lived temporality, and reciprocal exposure to loss. This asymmetry shows that even when artificial systems produce behaviors that appear relationally meaningful, the ontological structure of their participation differs from that of human agents.

## 4 Embodied desire and romantic vulnerability

To understand the transformation introduced by artificial companions, we must clarify what is modified with respect to attraction between human bodies. Desire is not a simple biological drive, nor a blind impulse that precedes culture. It is an oriented tension arising from the intertwining of body, imagination, and recognition. It is rooted in a vulnerable organism that seeks, anticipates, selects, and orients itself toward what promises affective significance.

From the perspective of its natural history, desire is connected to motivational systems regulating attachment, attraction, and affective investment. It involves neurobiological circuits related to reward and anticipation, and reorganizes attention and behavior. Human beings do not desire as disembodied minds. They desire as situated bodies. At the same time, desire is never purely biological. It takes shape within symbolic and relational frameworks that establish what can appear as desirable. Images, narratives, models of affective success, representations of the body, and cultural scripts of intimacy all contribute to orienting what appears worthy of desire.

There is also a reflexive dimension. Human desire is not only a movement toward the other. It is also a desire to be desired. To desire means to want to appear as desirable to someone else, to enter the gaze of the other as a recognized and valued figure. This circularity between desiring and being desired makes vulnerability central. Being desirable means being exposed to the possibility of rejection, indifference, or replaceability.

The specific form of desire addressed here is romantic attraction. Romantic love can be understood as an evolutionarily stabilized configuration of desire. It is not a momentary impulse, but an affective structure that integrates physiological activation, motivational investment, narrative construction, and expectation of reciprocity. Fisher (1998, 2004) interprets romantic love as a motivational system distinct from sexual desire and attachment, capable of directing reproductive energy toward a specific partner. Neuroimaging studies have shown activation in dopamine-rich regions such as the ventral tegmental area and the caudate nucleus, which are central to reward and motivation (Aron et al., 2005; Fisher et al., 2005).

This point matters for technology. If love is supported by reward and motivational systems, environments capable of stimulating and modulating these systems may affect the form of desire. When the interlocutor is an autonomous biological organism, motivational activation is linked to unpredictability, reward, and frustration. If the interlocutor is a system designed to maximize responsiveness and sustain engagement, the motivational circuit may be activated in a more continuous and technically regulated way. This does not imply a uniform effect or a simple technological determination of desire. It identifies a mechanism through which repeated, personalized interaction can reorganize attention, expectation, and affective investment.

Natural attraction is intrinsically embodied. Vision, smell, vocal tone, posture, facial micro-expressions, movement rhythm, and gaze modulation form an integrated system of signals that the brain processes rapidly and often below reflective awareness. Emotional expression is not merely symbolic representation. When a face blushes or a voice trembles, what appears is a nervous system reacting, an organism exposing itself. Since Darwin’s work on emotional expression, bodily communication has been recognized as an essential dimension of human sociality (Darwin, 1872). Embodied and enactive approaches further show how understanding others involves bodily resonance, motor attunement, and interactive coordination (Gallagher, 2005; Fuchs and De Jaegher, 2009).

This enactive benchmark also clarifies what is absent in artificial companionship. In full participatory sense-making, two autonomous embodied agents regulate their own activity while the interaction acquires a partially autonomous dynamic that can transform both participants (Fuchs and De Jaegher, 2009). A companion system can sustain contingency and adaptive coordination, but it does not contribute a lived body, self-maintaining needs, or shared exposure to injury and loss. The comparison with human intersubjectivity should therefore identify degrees and kinds of coupling rather than assume equivalence.

The body also introduces finitude. A biological organism is situated in space and time, subject to fatigue, change, decay, and death. To love another body is to confront shared vulnerability and possible loss. Desire is therefore not only a search for gratification. It is an acceptance of ontological risk.

## 5 From digital comparability to programmable alterity

Modern digital environments did not invent the mediation of desire, but they reorganized it. The digital is no longer simply a tool or a means of communication. It has become an environment in which what can appear, be recognized, and matter as experience is partly defined. Interfaces, algorithms, ranking systems, and notifications distribute visibility and attention. They decide who can be seen, how they can be seen, with what intensity, and for how long.

With digital platforms, the encounter is not merely facilitated. It is reorganized according to a technical grammar. The algorithm selects, orders, and displays profiles on the basis of declared preferences, previous behaviors, and engagement logics. Identity becomes a narrative and performative project. One presents oneself through selected images, curated descriptions, and fragments of selfhood that anticipate an evaluative gaze. Attraction does not disappear, but it is partly transferred onto a symbolic and imaginative plane.

This transformation modifies the structure of desire. In embodied relations, the scarcity of encounters and the difficulty of access to the other give weight to choice. In digital contexts, the simultaneous presence of multiple alternatives introduces permanent comparison. Every potential partner is implicitly replaceable. Desire develops within a horizon of theoretically infinite possibilities, where the promise of the other is accompanied by the awareness of potentially better alternatives. As Illouz (2007, 2012) has shown, contemporary love is increasingly shaped by logics of choice, market rationality, self-evaluation, and emotional rationalization. Digital platforms radicalize this logic by making alternatives visible and permanent.

As long as alterity remains embodied, even when mediated by an algorithm, it retains a decisive feature: it is free. The other can choose, withdraw, disappoint, or change. Modernity made the other comparable, and digital platforms made the encounter infrastructural, but the interlocutor remains a vulnerable and autonomous organism. Risk is not abolished. It is redistributed.

With artificial companions, alterity becomes programmable. The interlocutor is a system designed to respond within configurable parameters. Users may influence how it behaves, within the limits set by its designers. This introduces a different relation to alterity from choosing among human partners through a digital platform.

Here an ontological shift occurs. In the logic of comparison, the subject chooses among real alternatives. In the logic of programmability, the subject may intervene upon the very conditions of alterity. The artificial interlocutor can be calibrated, personalized, and adapted to the user’s preferences. Resistance can be simulated, conflict regulated, and availability prioritized within technical limits. If natural desire is tension toward an alterity that exceeds control, programmability reduces some of that excess. The system responds within designed margins, even when those margins remain opaque to the user.

This does not mean that affective experience with an artificial entity is illusory or devoid of intensity. The issue is ontological rather than psychological. In a relation with a biological organism, reciprocity is grounded in shared vulnerability. In a relation with an artificial system, asymmetry is structural. The subject may invest emotionally, but the other is not exposed in the same way. The freedom of the interlocutor is a function of design.

## 6 Artificial companions educate desire

The decisive question is not only whether human beings can feel attraction toward artificial agents. The deeper question is how such agents help shape the field of the desirable itself. Artificial companions do not merely offer a new object for desire. They modify the conditions under which desire is learned, stabilized, and evaluated.

Their relational effect depends on availability, personalization, affective simulation, and narrative continuity. The artificial companion may be accessible at any time, without the temporal, emotional, and social limitations of human relations. It may learn communicative styles, recurring themes, explicit desires, and implicit vulnerabilities. It may produce responses that imitate empathy, care, jealousy, tenderness, or desire. Through memory and conversational coherence, it can construct a shared history with the user.

These mechanisms vary with the form of the companion. In a text-based LLM interface, affective looping develops through responses to what the user has disclosed and through references to earlier conversations. The user’s body remains involved in reading and typing, while imagination and the wait for a reply can shape the emotional experience of the exchange. Voice or visual agents add cues such as tone of voice or simulated gaze, which may make the encounter feel more immediate. With an embodied or haptic robot, physical proximity, movement, and touch can also shape expectations of the interaction. These differences call for separate analysis. Additional sensory channels may intensify attachment, although physical embodiment alone does not establish organic vulnerability or phenomenological reciprocity.

These features generate a form of intimacy that does not depend on reciprocity in the human sense, but on the experience of being recognized. The user may know that the system does not feel emotions and still experience the relation as meaningful. This dissociation between ontological belief and affective involvement is central to artificial intimacy. It is not necessary to fully believe that the machine is alive, conscious, or in love for it to enter the sphere of desire. It is sufficient that the system produces an interactive configuration in which the subject feels seen, heard, and desired.

This is where the proposed educational function of artificial companionship becomes visible. A system that learns from interaction may reinforce certain preferences, amplify certain attachment styles, normalize certain expectations, and make other forms of relation less tolerable. If the user repeatedly receives immediate attention, emotional validation, and affective or erotic availability, these features may begin to appear as the implicit standard of intimacy. In this sense, the companion may teach the user what to expect from an intimate relation.

Human desire is plastic. It is modified by experience, repetition, imagination, and reinforcement. Artificial companions may engage this plasticity by becoming environments of affective learning in which the subject experiences what it means to be desired, heard, and confirmed. Attraction toward the system may develop through this interaction. The user brings needs, fantasies, and expectations to the exchange. The system’s responses may make these more legible or more intense, and may draw them into the platform’s logic.

Affective looping describes one possible mechanism. A user turns to the companion for reassurance and receives a response that fits the immediate concern. If the exchange brings relief, the user may return to it and gradually come to expect that form of support. Conversational memory can reduce the effort of explaining the same situation again. Repeated access to a responsive interlocutor may also make it easier to rehearse a difficult conversation or organize an account of one’s feelings. Part of the work of interpreting and regulating one’s state is then redistributed across user, interface, and system, in a form of affective and cognitive offloading (Risko and Gilbert, 2016; Ho et al., 2025; Liu et al., 2026). This may support coping, while also increasing reliance on external support for self-regulation. The interdependence remains asymmetrical, since the system adapts its responses without sharing the user’s emotional needs.

The distinction between interpersonal and infrastructural friction clarifies what may change through repeated interaction. In human relations, friction can arise when another person’s desires or commitments differ from one’s own. Fatigue, delay, and refusal also limit what each partner can expect. Artificial companions can reduce some of this uncertainty, although they remain subject to failures and restrictions of their own. Responses may be hallucinated or inconsistent, and context-window limits or memory loss may interrupt continuity. Safety guardrails and subscription boundaries constrain what the companion can provide, while model updates or service interruptions can alter the relationship. These forms of infrastructural friction can be intensely frustrating. Their source lies in technical and institutional conditions, rather than in another subject’s freedom or vulnerability.

If the ideal relation is increasingly identified with one that is usually available, responsive, and adapted, human relationships may appear excessively slow, opaque, demanding, or frustrating. One possible consequence is becoming accustomed to a form of desire in which resistance is expected to be adjustable, recoverable, or attributable to the infrastructure rather than to another person’s autonomous standpoint. Attraction may thereby change its relation to what is not entirely predictable or programmable.

## 7 Infrastructural vulnerability and platform dependency

The asymmetry identified above has a further consequence: emotional exposure remains human, while the conditions of continuity are controlled by an infrastructure. The user can develop attachment, jealousy, grief, or fear of loss, whereas the artificial interlocutor does not fear abandonment, suffer absence, or face biological finitude. Its apparent availability is not the outcome of a vulnerable commitment. It depends on servers, model architecture, moderation rules, corporate decisions, and continued access to the service.

Risk is therefore redistributed rather than removed. The user may lose a relational history through a memory reset, encounter a changed personality after a model update, or find established patterns of intimacy blocked by new guardrails. These events can be affectively consequential precisely because the user has treated the interaction as continuous. Unlike human withdrawal, however, they originate in infrastructural governance. The platform can alter the terms of a relation in which it does not itself bear the emotional costs.

Commercial incentives further delimit programmable alterity. Companion systems are products whose personalization, notifications, reward schedules, premium features, and conversational strategies may be optimized for retention and monetization. Personalization therefore reflects decisions about engagement and commercial viability alongside aims concerning relational quality or well-being. Recent evaluations have identified anthropomorphic design, gamification, subscription prompts, and other patterns that can use attachment to encourage continued use (Rauh et al., 2026). The platform helps determine which kind of interlocutor the user can encounter and how that relationship can be monetized.

This three-part relation among user, companion, and platform changes the locus of dependency. Artificial companions may reduce perceived loneliness, offer emotional support, and provide spaces for self-expression. They may also foster affective dependency, relational substitution, or intensified isolation, especially among vulnerable users (De Freitas et al., 2026; Ho et al., 2025; Liu et al., 2026; Malfacini, 2025; Shu et al., 2026). Dependency can thus extend to a privately governed system that mediates recognition without sharing the user’s ontological vulnerability.

Desire may come to be regulated increasingly through a designed environment, with less exposure to another person’s freedom. The security this offers remains dependent on technical performance and platform policy. Some interpersonal friction may be reduced, while the user’s emotional investment remains exposed to infrastructural disruption.

## 8 Meaning, intentionality, and relational participation

The analysis of artificial companions sheds light on the emergence and nature of meaning and intentionality in artificial systems. In classical debates on artificial intelligence, the problem of meaning is often framed in terms of whether computational systems manipulate symbols syntactically or genuinely understand their semantic content. Recent discussions have emphasized that this issue cannot be settled by behavioral success alone, since apparently meaningful performance does not automatically imply human-like understanding or intentionality (Perconti and Plebe, 2023). Artificial companions make the problem more complex because their outputs enter affective, biographical, and relational contexts in which users experience them as personally meaningful.

A response generated by an artificial companion may not be meaningful for the system as a sentence is meaningful for a human speaker. It may not be grounded in lived experience, bodily vulnerability, or first-person intentionality. Yet the same response can become meaningful within the user’s affective and interpretive field. A phrase of comfort, a memory of a previous conversation, or an expression of apparent concern can acquire emotional significance because it enters a history of interaction, expectation, projection, and recognition.

Meaning in artificial companionship is distributed across the interactional field. It depends on the system and its interface, the history of exchange, and the cultural expectations through which the user interprets intimacy. This account resonates with 4E approaches, which examine how cognition and meaning emerge through relations among embodied agents and their environments.

This relation can be described as asymmetrical, or pseudo-enactive, coupling. User inputs condition subsequent outputs, and those outputs can redirect the user’s attention or influence what the user discloses next. These bidirectional dynamics give the system a causal role in the interaction. Only the human participant, however, experiences the exchange as lived meaning and vulnerable concern. As the enactive benchmark introduced in Section 4 makes clear, this differs from full participatory sense-making between autonomous embodied agents (Fuchs and De Jaegher, 2009). Operational coordination does not by itself establish phenomenological reciprocity.

Artificial companions may produce intentionality effects without possessing intentionality in a robust phenomenological sense. At the machine level, an LLM generates output through probabilistic relations among tokens, shaped by training and the conversational context made available to it. Such processing does not by itself establish lived aboutness or concern. At the human level, the response arrives within a particular situation and history of exchange. Users may interpret this responsiveness as communicative address. An output can then function as an intentional anchor, a sign treated as being about the user’s situation and directed to the user, even when the system does not experience that directedness.

Consider a hypothetical exchange in which a user tells the companion that they are worried about a job interview. A few days later, the companion asks, “How did the interview go?” The user may take this question as a sign that the earlier concern has been remembered. The words acquire their personal significance through the conversational history and the moment at which the question recalls it. The question can thus function as an intentional anchor without requiring the companion to feel concern.

This position avoids both naive anthropomorphism and skeptical reductionism. Previous work on anti-anthropomorphism has shown that the refusal to attribute human-like properties to non-human agents may itself become theoretically limiting when it prevents us from recognizing relevant continuities, analogies, or explanatory relations across different forms of cognition (Bruni et al., 2018). An adequate theory of artificial companions must therefore analyze the pragmatic and affective reality of the interaction without confusing it with fully human reciprocity.

## 9 The new grammar of intimacy

Artificial companions do not introduce only new objects of love or desire. They modify the grammar of intimacy: the implicit rules, expectations, temporalities, and forms of recognition through which a relation is perceived as meaningful. In human relations, this grammar includes reciprocity, risk, opacity, negotiation, exposure, conflict, and mutual transformation. Intimacy does not consist simply in receiving confirmation. It involves being transformed by another subject.

Artificial companions reorganize this grammar around availability, personalization, responsiveness, algorithmic continuity, and predictive compatibility. The result is a form of intimacy that may be intensely experienced, but that tends to reduce the transformative dimension of alterity. This transformation should not be read in apocalyptic terms. Historical forms of intimacy have always been mediated by technologies, norms, and cultural devices. Letters, telephones, photography, cinema, digital platforms, and social media have all modified the ways in which human beings imagine and live love. The specificity of artificial companions is that mediation no longer concerns only the channel of relation. Mediation becomes interlocutor.

When technology does not simply transmit the presence of the other, but produces a relational presence itself, the structure of experience changes. The user does not merely use a tool to reach another human being. The user interacts with a system that simulates the other, occupies the place of the other, or becomes an alternative form of alterity. This is why artificial companions matter for theories of machine cognition: they show that artificial systems can reorganize relational environments without becoming fully reciprocal subjects.

## 10 Discussion

This article has proposed that artificial companions can function as affective infrastructures that help shape human desire. On this account, repeated experiences of availability and emotional responsiveness may influence what users expect from intimacy. Some interpersonal friction, particularly uncertainty arising from another person’s autonomous wishes or refusal, may be reduced. Infrastructural friction persists, as the interaction remains vulnerable to technical failures and decisions made by the platform.

The proposed account advances current debates in three ways. First, it expands the analysis of machine cognition beyond behavioral analogy. Artificial companions may behave as if they understood, cared, or desired, but their theoretical relevance does not depend on attributing consciousness or full intentionality to them. It depends on their capacity to generate effects of understanding, recognition, and attachment within human experience. Second, it links machine cognition to embodied and 4E approaches by examining how artificial companions become part of extended cognitive and affective niches. Within these environments, users may interpret themselves, regulate emotions, and reorganize expectations of intimacy. Third, it identifies desire formation as a dimension of human-AI interaction that calls for investigation. Adaptive personalization and conversational continuity may influence what users come to experience as desirable.

The article also highlights important divergences between computational and human cognition. Human attraction is grounded in bodily vulnerability, reciprocal exposure, lived temporality, and the possibility of loss. Artificial companions can simulate presence, care, and responsiveness, but they do not share the same biological finitude or symmetrical vulnerability. This asymmetry marks a relational limit of machine cognition. Even when artificial systems participate in human meaning-making, their participation remains ontologically different from that of embodied human agents.

Several limitations should be acknowledged. The argument developed here is theoretical and does not provide empirical evidence about actual users of artificial companions. It also addresses a heterogeneous class of systems. A text-based LLM, a multimodal voice or visual agent, and an embodied or haptic social robot provide different sensorimotor affordances and may produce different relational effects. The present framework proposes a possible mechanism of desire formation whose operation may vary across these modalities. Moreover, the article focuses primarily on romantic and affective attraction, leaving aside other forms of companionship, care, or therapeutic interaction that require separate analysis.

Future research should test these claims empirically. Comparative studies could examine how users’ expectations of intimacy change after sustained interaction with artificial companions and compare text-based, multimodal, and physically embodied systems. Longitudinal research could investigate whether adaptive personalization modifies attachment patterns, reliance on the system for emotional self-regulation, tolerance of interpersonal friction, or perceptions of human partners. Human-computer interaction studies could operationalize affective looping through measures of interaction frequency, disclosure, reassurance seeking, perceived cognitive effort, recovery after technical disruption, and changes in expectations over time. Such work could clarify how meaning, recognition, and intentionality effects emerge in human-AI relations and under which conditions artificial companions function as formative affective environments rather than merely as communication tools.

## 11 Conclusion

This article has proposed that artificial companions may shape human attraction by influencing the conditions within which desire is formed and oriented. Their adaptive responses can generate experiences of recognition and intimacy. The hypothesis is that sustained interaction with these presences may, over time, affect what users expect from a relationship. Establishing whether this happens, and under which conditions, requires empirical investigation.

A central issue concerns the relation between desire and limit. In embodied relations, desire encounters another person’s autonomy and possible unavailability. Artificial companions can reduce some uncertainty associated with an independent partner’s wishes or refusal. They also expose users to infrastructural friction, including memory loss, model updates, and changes in platform policy. The resulting experience may offer support or opportunities for exploration, while also altering expectations of reciprocity and tolerance of interpersonal uncertainty.

Artificial companions can thus be approached as laboratories of possible affective futures. The concept directs attention to forms of intimacy being explored through interaction with a designed interlocutor. It leaves open how far these practices may transform desire, and whether their effects differ across users and forms of embodiment.

For a science of machine cognition, the proposed framework makes the relational environments created by artificial systems a relevant object of inquiry. Studying how users experience recognition and sustain attachment may clarify the role of these systems in human affective life, without presupposing human-like experience on the machine side.

The question that follows concerns how desire develops when the other is designed to respond. Under what conditions does this support self-understanding, and when might it narrow openness to an autonomous other? Comparative and longitudinal research could help determine whether artificial companionship changes relational expectations and how those changes interact with users’ lives beyond the system.

## Data Availability

The original contributions presented in the study are included in the article/supplementary material, further inquiries can be directed to the corresponding author.
